# Exploring Chinese secondary students’ behaviors towards online homework based on the UX model: Does homework completion relate to academic performance?

**DOI:** 10.1016/j.heliyon.2024.e40472

**Published:** 2024-11-22

**Authors:** Liu Chen, Su Luan Wong, Shaoning Zeng, Shwu Pyng How

**Affiliations:** a Department of Science and Technical Education, Faculty of Educational Studies, Universiti Putra Malaysia, Malaysia; b Yangtze Delta Region Institute (Huzhou), University of Electronic Science and Technology of China, China

**Keywords:** Academic performance, Online homework completion, Student learning, The UX model

## Abstract

The benefits of online homework are widely acknowledged for student learning. Numerous studies have provided evidence supporting the notion that the completion of an adequate amount of traditional homework is associated with enhanced academic success. However, there are inconsistent conclusions on the effect of homework completion on academic performance among the students who were exposed to online homework, particularly in China. In order to explore the behaviors of Chinese secondary students towards online homework, a correlational analysis is employed to determine the relationship between homework completion and academic performance among 103 students from China. Findings suggest a positive yet moderate association between online homework completion and academic performance. A follow-up qualitative interview with 10 purposefully selected students, based on the UX model by Topolewski, Krawczyk, Pallot & Huotari (2020), reveals that the cognitive and emotional factors are most influential on their behaviors towards online homework. Further studies are recommended to delve into the factors influencing students’ intention to engage with online homework. Additionally, limitations of this study are outlined.

## Highlights

• The impact of online homework on Chinese secondary students' academic performance has been under-researched.
• A positive but moderate correlation exists between online homework completion frequency and academic performance among 103 students.
• Follow-up interviews with 10 studentsbased on the UX model , showcognitive and emotional factors affect their learning behaviors.
• It provides a reference for investigating student-side factors that predict technology-based learning behaviors.

## 1 Introduction

Homework is a common educational activity for students to complete outside of regular school hours [1]. Defined as homework assigned via an online software system that functions by synchronizing learning activities, providing prompt feedback, analysing learning acquisition, and customizing individual learning plan [2], online homework is widely integrated into the instructional process due to the technological advancement [3]. The benefits of this facilitator for student learning, including customized and hierarchical homework design, immediate feedback, automatic grading, and monitoring systems, is also well documented by researchers [[3], [4], [5], [6]]. Acknowledging that the advantages of homework has been recognized by students and teachers, identifying the characteristics of homework, such as frequency, purpose [7], and format [8,9], to boost the engagement of students in homework assignments becomes significant [10].

Regarding the traditional format, studies have demonstrated that homework completion is indeed associated with academic achievement [[11], [12], [13], [14], [15], [16]]. However, research on the correlation between homework completion and academic achievement of the students who were exposed to online homework are still challenging to ascertain [[17], [18], [19]]. Particularly, existing studies are commonly targeted at the college-level students who accepted online homework in terms of homework completion [19]. The introduction of online homework is beneficial with the consideration of the assessment of student learning for different subjects in different contexts throughout schooling [20,21]. Therefore, engaging in educational research within the context of K12 schooling holds considerable importance in order to fully comprehend the advantages of online assignments for student learning [3,22].

Owing to the rapid iterative update and the use of technology, online homework has emerged to supplement the teaching and learning process in China. In the field of K12 education, many online homework systems, such as 17zuoye, ETS, and Ekwing, have been promoted by the government and companies to fulfill the requirements of teachers and students [23]. The emergence of online homework seems to offer an alternative to supplement the defects of traditional homework (e.g., delayed feedback and irregular assessment) for teaching and learning. Nevertheless, with a Confucianism heritage culture and known as an examination-driven orientation for Asian education, small proportion of Chinese secondary students would like to do homework online to achieve academic performance [24]. Some are reluctant to expose to this learning facilitator or decline to accept it for learning [25]. What is worse, informed studies focused on secondary school students in the Chinese context are also insufficient [22]. Hence, it is imperative to address this gap.

Gaining insight into users' experiences with technology enables the identification and potential anticipation of their behaviours [26]. Researchers have investigated the consequence of using technology among users and their inclination to adopt it through their experiences in using the technology [27,28]. Evidently, data about the students' experience in using online homework can provide information to help understand their learning behaviors, leading to better enhancement of student learning [29]. In turn, such data can also be used to help improve students' persistence and retention in online homework [30,31]. Hence, understanding students’ experience in using online homework may account for the relationship between homework completion and academic performance among students.

In this case, the current study conducted a follow-up interview with purposefully selected students to understand their experience in using the system. Relevant reports collected from the students were investigated based on the UX model by Topolewski, Krawczyk, Pallot & Huotari [32]. This study represents a fresh endeavour aimed at investigating the experiences of Chinese secondary students (students from junior and high schools) in this particular context. Considering the significance of online homework in facilitating learning consolidation, it is crucial to comprehend the correlation between the completion of homework and academic achievement, as well as the factors that influence students’ behaviour towards online homework for learning. By tailoring assignments to individual students, modifying teaching strategies, and exploring more effective methods to enhance student learning, educators can optimise the benefits of online homework.

## 2 Literature review

As a common instructional technique, homework demands students to devote time, energy, and effort to complete it [33]. Online homework, as a format of homework delivery, also requires similar devotion from students. Homework completion, often defined as the frequency of finishing homework assignments, has been a variable examined in studies exploring its relationship with academic achievement [7]. Besides, data show that students’ experience in doing online homework also contributes to understanding their behaviors [26].

### 2.1 Studies on online homework completion and academic achievement

The extant literature on the relationship between homework completion and academic achievement of students exposed to online assignment reflects different and often contradictory findings. Parker & Loudon [2] found a positive relationship between online homework completion and the students’ final grade in an introductory organic chemistry course due to the immediate feedback provided by the system. Kevin [17] also found that students in a large introductory chemistry class who completed most or all online homework performed better than those who did not. Conversely, Gascoigne [34] noted that while post-secondary language students who skipped online homework still met their expected exam grades, they failed to achieve A or A+ marks, likely due to low self-efficacy. Cosio & Williamson [19] investigated the timing of homework completion in general chemistry but found no clear correlation with academic performance. For high school students, Evans [9] demonstrated that a high perceived usefulness of online homework, particularly due to instant feedback, was strongly linked to their intention to complete and reattempt assignments.

Completing homework is believed to enhance students’ conscientiousness across various academic levels [7,15,35]. However, the correlation between online homework completion and academic achievement among K12 students in China remains ambiguous. Cheng [36] reported positive attitudes and benefits in language learning among elementary students who completed online homework. Similarly, Wu, Li, Zhu, Yang, Bai, Zhao & Yang [37] found a significant positive association between online homework completion rates and learning achievement among fourth-grade students, moderated by prior academic achievement and parent-teacher partnerships. Underpinning the zone of proximal development and constructivism theory, Zhang [38] linked the quality of homework completion to academic achievement in junior school students due to improved self-directed learning and increased interest in math. In contrast, Huang [39] observed that while junior school students developed some interest in English through personalized online assignments, their academic performance showed insignificant improvement due to negative attitudes towards the assignments.

According to the findings of the aforementioned studies, there appear to be inconsistencies in homework completion and academic achievement among Chinese students exposed to online homework. Apart from the functions provided by online homework, such as instant feedback and individualized content design, it suggests that other potential variables particularly concerning student-side factors (e.g., attitude) may account for this association in the Chinese educational context. As a result, it is critical to explore the relationship between online assignment completion and academic success as well as related influential factors so as to gain a thorough grasp of this topic.

### 2.2 Studies on students’ experience in using online homework

A thorough understanding of the user experience with technology is crucial for comprehending their behaviors. Users' subjective feelings about technology may even determine the acceptance of technology [26]. To the context of online homework, various studies have reported on students’ perceptions and interactions with the system.

Many students appreciate online homework due to its perceived usefulness. Yu, Lai, Liew, Tan & Noum [29] found that 58.5 % of students gave favorable comments on online assignments, citing the success of system in delivering learning outcomes. Conversely, students may reject online homework if they do not see improvements in their learning. Alawashi & Abu-Ayyash [40] noted that though the system enhanced the enjoyable learning experience, it did not significantly affect academic performance, leading to reluctance among students.

Research also indicates that enjoyment and satisfaction are key to embracing online homework. Maxwell, Smoker & Stites-Doe [30] found that users motivated by satisfaction with the system are more likely to use it. Metwally, Chang, Wang & Youself [31] reported that gamification in assignments enhanced enjoyment and interest, although it did not guarantee improved academic performance of students.

To sum up, studies focused on college and high school levels have demonstrated the mixed impact of homework completion on student learning. Their prior experience with online schoolwork may also help to explain their actions towards the system. Unfortunately, there is insufficient evidence on the academic achievement of secondary students based on the actual setting of online homework, particularly in China. To understand Chinese secondary students' behaviors towards online homework, this paper is proposed to examine the correlation between academic achievement and online homework completion. Furthermore, students’ experiences with online homework are also being investigated from the perspective of the UX model by Topolewski, Krawczyk, Pallot & Huotari [32] to gain insights into factors influencing their motivation to complete assignments. In this study, academic achievement is defined as the scores of the final exam for the semester. Online homework refers to homework assigned by instructors to students for English learning via the system. The research questions that guided this study are as follows:

RQ1 Is there a relationship between online homework completion and academic achievement of Chinese secondary school students?

RQ2 What are Chinese secondary school students' experiences with online homework?

## 3 Theoretical framework

**Fig. 1:**
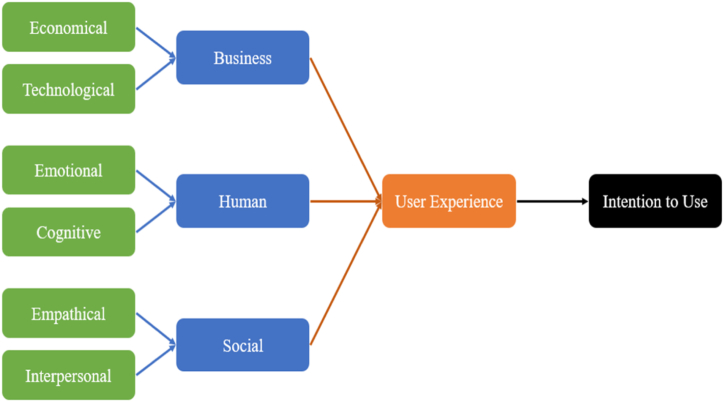
User experience model by Topolewski, Krawczyk, Pallot & Huotari (2020).

According to Pallot, Kalverkamp, Vicini, Trousse, Vilmos, Furdik & Nikolov [41], user experience is a broad and multidimensional construct, representing social and empathical experiences among users when using a product. The concept is commonly recognized to consist of both objective and subjective elements. In this study, the UX model from Topolewski, Krawczyk, Pallot & Huotari [32] was adopted as the theoretical framework, because this model not only empirically evaluates user experience but also explores the impact on potential adoption of technology among users. As shown in Fig. 1, this model embraces three dimensions to evaluate user experience, namely human dimension, social dimension, and business dimension. Emotional and cognitive factors are included in the human dimension. Empathical and interpersonal factors are included in the social dimension. While economic and technological factors are in the business dimension.

The authors empirically demonstrated that almost 71 % of the variability of the user intention to use technology can be explained by user experience. In terms of human dimension, emotional factors refer to the degree of attractiveness, enjoyment, as well as fulfilments that users experience in using technology, while cognitive factors refer to the degree of comprehensiveness, engagement, as well as meaningfulness that users have obtained from technology. For social dimension, empathical factors refer to the degree of attentiveness, helpfulness, as well as responsiveness that users can offer to others when using technology, while interpersonal factors are the degree of collaborativeness, communicativeness, as well as confidence that users perceive in using technology. Regarding business dimension, economic factors are defined as the extent of entertaining, pleasantness, as well as productivity that users experience when using technology, while technological factor are the extent of novelty, efficiency, reliability, as well as user-friendliness that users experience in using technology.

## 4 Methodology

A cross-sectional research design was employed in this study. A correlational analysis was conducted to assess the relationship between homework completion and academic achievement of the students who were exposed to online homework for English learning. Additionally, according to Creswell [42], exploratory elements are helpful for researchers to understand information and carry out follow-up studies. Accordingly, a follow-up qualitative interview was also applied to determine the students’ experiences in using online homework.

A convenience sampling method was employed in this study. The invited students were 103 eighth graders taught by the same instructor from a public secondary school of Huizhou, Guangdong, China, including 51 students from Class 1 and 52 students from Class 2. All students were exposed to an online homework system to complete the English homework assigned via the instructors voluntarily for one semester. The time allotment for students to complete this task was 60 min per assignment with 65 items in one week. The items consisted of 30 listening questions, 10 grammar choices, 10 cloze tests, and 15 reading comprehension questions. At the end of the semester, all students completed the semester exam with a total point of 100, which was taken as the post-test of student learning. The use of the 100-point scoring system to evaluate students' academic performance is widely employed in grading practices [43], hence this study also adopted this rubric to evaluate students’ academic performance due to its clear and comparative measure. The content of exams was all based on Compulsory Education English Curriculum Standards in China [44]. Besides, the assignment question types were identical to satisfying the assessment requirement of the semester exam with a multiple-choice option setting. All students had only one chance to complete homework online at the scheduled time in this study. To secure the reliability of the exam scores, the semester exam (post-test exam) conducted by the Huicheng District Education Bureau of Huizhou was adopted and adapted to evaluate the academic achievement of students in English learning. The content of the exam was aligned with the national curriculum standards to ensure consistency and validity in evaluation.

The frequency of online homework completion was measured. In this study, only all items included in each assignment had been finished that can be taken as completing one assignment. The same teacher instructed students the assignments and the number of online homework was arranged by the official academic week. There was a total of 19 weeks of school teaching in the first semester of the 2022/2023 academic year, including 17 weeks of lecture teaching and 1 week for the mid-term exam and 1 week for the final exam. Hence, a total of 17 online homework was assigned by the teacher. Each student's homework completion frequency was calculated regularly by the system, then double-checked and summed up by the researchers at the end of the semester.

A semi-structured interview protocol underwent revision by two experts with backgrounds in education was used to investigate students’ experience in using online homework. Students were invited to participate in the interview with the option of withdrawing. In the current study, the interviewees were purposefully selected for the interview session by the researcher. The considerations of the interviewees for the study included age, gender, extreme cases of low or high academic performance, and frequency of homework completion. Besides, qualitative interviews may be conducted with a few individuals [45]. In this case, 10 students were purposefully selected from the two classes, each with five students respectively. They were interviewed individually by the researcher. Before starting the interview, a pilot test was conducted with two students to ensure the clarification of the questions as well. According to Guest, Bunce & Johnson [46], data saturation can often be reached relatively quickly, depending on the homogeneity of the sample and the complexity of the questions being asked. Besides, if the interview session is well-structured and focused on specific questions, the shorter length of interviews can still be effective [47]. Therefore, each interview, lasting about 10–15 min, was conducted in Chinese and audio-taped. Concerning the research ethics, this study has been approved by the Research Ethics Committee for Research Involving Human Subjects at Universiti Putra Malaysia, and the principals of the participating school. All students agreed to sign an informed consent form prior to the study. After each interview, the recorded data were transcribed verbatim and immediately coded by two authors. In the event of uncertainty or disagreement during coding between two authors, the third author refereed the discrepancies. Via thematic analysis technique, sub-categories and themes were identified.

## 5 Data analysis and results

### 5.1 Correlational analysis

In this study, 103 students instructed by the same teacher participated in the investigation to complete English homework online. There were 43 female students and 60 male students, respectively. For homework completion, the frequency was recorded between 0 and 17. The descriptive statistics of frequency of online homework completion was *M* = 9.40, *SD* = 4.03 (see Table 1). As indicated in Table 2, 1.9 % of the students failed to complete one assignment. Conversely, 2.9 % of the students managed to complete all assignments. Fortunately, 58.3 % of the students accomplished more than half of the given assignments. However, 12.6 % of the students completed assignments fewer than 5 times.

**Table 1:** Descriptive analysis of scores and frequency of online homework completion.

	N	Mean	*SD*	skewness		kurtosis	
				statistics	Std. error	statistics	Std. error
Scores	103	49.21	22.63	.152	.238	−1.36	.472
Frequency∗	103	9.40	4.028	−.275	.238	.300	.472
Valid N	103						

Frequency∗ = Frequency of online homework completion.

**Table 2:** The distribution of the frequency of online homework completion.

Frequency of homework completion	Accumulated percentage (%)
0	1.9
1–4	10.7
5–8	26.2
9–12	37.9
13–16	20.4
17	2.9

The descriptive of scores was *M* = 49.21, *SD* = 22.63 (see Table 1). Among the students, 10.7 % of students achieved over 80 scores in their final exams. However, 7.8 % of students failed to reach even 20 scores in the same exam. The majority of students scored between 21 and 40. Regrettably, over two-thirds of students were unable to attain a passing score of 60, as presented in Table 3.

**Table 3:** The distribution of exam scores.

Scores	Accumulated percentage (%)
0–20	7.8
21–40	34.9
41–60	22.3
61–79	24.3
80–100	10.7

Skewness and kurtosis serve as indicators for testing the normality of data distribution. Typically, the values of skewness and kurtosis from −1.00 to +1.00 are deemed excellent while those between −2.00 and + 2.00 are generally acceptable in most cases [48]. As presented in Table 1, the skewness and kurtosis of scores and frequency of online homework completion were within the acceptance range. Consequently, both variables examined in this study exhibited with normality distribution.

**Fig. 2:**
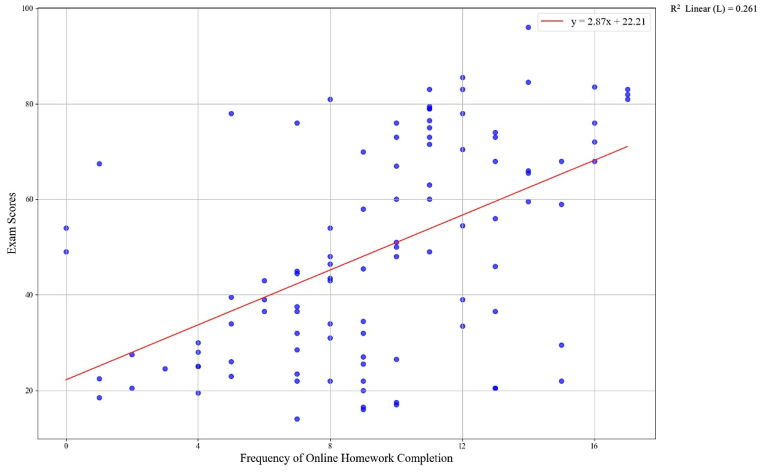
The scatterplot of the correlational relationship.

In order to examine the relationship between online homework completion and academic performance among students, Pearson product-moment correlation was conducted. Based on the calculation of the Pearson correlation coefficients, the data unveiled that the frequency of online homework completion and academic performance (post-test scores) was positively correlated, *r* (103) = .511, *p* = .000 (*p* < .05), as shown in Table 4. Students who frequently did online homework tended to get higher post-test scores, as illustrated in Fig. 2. Despite the positive relationship observed between online homework completion and academic achievement of students, the strength of this correlation was considered moderate [49]. Additionally, the statistics revealed that the variable of online homework completion accounted for approximately 26.1 % of the variance in the post-test scores of students who used the online homework system.

**Table 4:** Correlations between scores and frequency of online homework completion.

		Scores	Frequency^a^
Scores	Pearson Correlation	1	.511^b^
	Sig. (2-tailed)		.000
	N	103	103

a Frequency = frequency of online homework completion.

b Correlation is significant at the.01 level (2-tailed).

### 5.2 Interview reports

In this study, 10 purposefully selected students were interviewed, identified, and assigned numbers 1 to 10. The qualitative interview data were transcribed, coded, and analyzed. Based on the user experience model by Topolewski, Krawczyk, Pallot & Huotari [32], efforts were made to reduce the number of codes by combining related categories to form a hierarchical coding scheme [50,51]. Through the inductive process, the researchers discussed, verified, and confirmed the coding categories. Subsequently, three categories emerged from the analyses, including human dimension, social dimension, and business dimension, as presented in Table 5. For confidentiality, the 10 students were referred to in this paper as S1-S10.

**Table 5:** The emerging themes are extracted from the interview transcript.

SS Identity	Theme						Behavioral Intention
	Human dimension		Social dimension		Business dimension		
	Cognitive factors	Emotional factors	Empathical factors	Interpersonal factors	Economical factors	Technological factors	
S1	not enhance AP^a^	interest, like				low speed, server standby	continue using
S2	enhance AP	enjoy	feedback; helpful to others			easy-to-use	continue using
S3	enhance AP	like	feedback; communication	parents and teachers	interesting	easy-to-use	neutral
S4	enhance AP	interest	feedback; communication	classmates		internet speed and server standby	neutral
S5	enhance AP	dislike		parents and classmates	boring		not intend to use
S6	enhance AP	like				server standby and internet speed	reject to use it
S7	enhance AP	like and enjoy	ease to communicate	parents	interesting	easy-to-use	prefer to use
S8	enhance AP	like and interest				internet speed, server standby	continue using
S9	enhance AP	enjoy		peer and parents		server standby, internet speed	continue using
S10	not enhance AP	dislike		parents	unattractive	internet speed	not intend to use

a SS = students, AP = academic performance.

As shown in Table 6, the theme that most frequently mentioned among the interviewees was human dimension (N = 20), followed by business dimension (N = 13), then social dimension (N = 10). Besides, the frequency of cognitive factors was the same as emotional factors (N = 10). Technological factors (N = 9) also emerged frequently among the interview data.

**Table 6:** The frequency of themes emerging in the qualitative data.

Theme	Human dimension		Social dimension		Business dimension	
	Cognitive factors	Emotional factors	Empathical factors	Interpersonal factors	Economical factors	Technological factors
Frequency	10	10	4	6	4	9
In total	20		10		13	

#### 5.2.1 Theme 1: cognitive and emotional factors in human dimension

Regarding cognitive factors, perceived usefulness is commonly acknowledged among the interviewees. The findings revealed that a majority of students (n = 8) admitted that the online homework system provided them with great benefits, particularly in terms of enhancing their academic performance. The format of homework delivery enabled them to master the coursework and acquire a deeper understanding of related subject knowledge. With the support of this system, they were capable of answering correctly in the exam and obtained better academic performance. For example, S4 emphasized: “*… it is with the help of the system that I find my learning ability has been enhanced. I not only can firmly keep in mind the coursework knowledge* via *online homework, but also get to know more related information* via *system. I believe that using this system could enhance my academic performance* …”.

Besides, two students stressed that they did not get an enhancement in their academic performance despite using this system to complete their homework. Consequently, they harbored some doubt regarding whether this system may actually assist them in achieving the expected level of academic performance. For S1, she expressed: *“… this system is helpful for me to complete homework as quickly as possible. To brief, it becomes much easier and simpler for me to complete homework with the related information* supported *by the system. However, I do not find its effect on my academic performance obviously. I am expected to gain progress* via *using the system. In fact, it does not come true in my exam …”.* For S10, he shared: *“… I become familiar with the course content with the help of online homework, and I also have a chance to improve my communication ability. But it does not enable me to get the expected higher scores in the exam …”.*

In relation to emotional factors, the findings showed that approximately half of the students confirmed experiencing positive emotions while engaging in doing online homework. Especially, students frequently mentioned that they were interested in doing online homework, particularly when the system praised them for excellent homework submissions. Moreover, many students also expressed their enjoyment of using the system for learning. For example, S1 said: *“The interesting assessment makes me interested in doing the online homework”.* S7 also expressed a positive attitude towards the system: “*I really prefer to doing homework* via *the system. When I am using the system, I feel really relaxed. I do not crush any pressure in using the system. It is a really simple, relaxing, and enjoyable learning facilitator*”.

#### 5.2.2 Theme 2: empathical factors and interpersonal factors in social dimension

In terms of empathical factors, helpfulness was mainly mentioned in the interview. Students expressed that the system enabled them to become confident and courage to communicate with others, even helped others. For example, S2 expressed: *“Thanks to the practice* via *the online homework for my oral English, I helped a foreigner order a coffee in a coffee shop when I was travelling in Taiwan”.* S3 also commented: “*I do not feel embarrassed any more when my instructor asks me to answer questions after I use the system for learning consolidation.”* S4 and S8 also claimed the alike opinions on this topic.

For interpersonal factors, the results indicated that completing online homework generally required parental supervision. Many students reported that they might play computer games, watch short videos, or surf the internet if they got the chances. Though they felt it was not good to do so, they cannot control themselves to focus on their homework. For example, S10 mentioned: *“… When I am doing online homework, I always cannot help doing something that is not associated with my study … I need my parents supervise me or company me when I am doing online homework* via *mobile phone …”*. In addition to parental supervision, students also mentioned that they had to require their parents' assistance while doing homework. This was due to disruptions caused by their younger siblings, which affected their concentration on their assignments. For example, S9 explained: *“… my little brother would sit beside me and then suddenly yell out or kick the keyboard. Hence, I have to ask my parents to take care of my little brother …”*.

#### 5.2.3 Theme 3: economical factors and technological factors in business dimension

With regard to economical factors, only two students claimed that the system was interesting enough to arouse their interest in using it to complete homework. For instance, S7 commented that: “*The content was interesting. Via this system, I can complete the homework without distractions* … *it is really enjoyable and interesting for me to do homework in such relaxing and simple context …”.* What is more, a few students showed dislike towards the system. As S5 said: “*I do not have any enthusiasm to do homework* via *the system anymore. I am impatient to do such boring and repetitive homework*”.

Concerning technological factors, the results showed that the design of the system encouraged students to use the system. For example, S3 expressed: *“In fact, I do not encounter any difficulties in using the system when I am doing homework. If there is a problem, I will find customer service and settle down the problem as soon as possible”.* S2, and S7 claimed the similar opinions towards this topic as well. However, the instability of the online homework system frequently led to a black screen and server standby. Despite these challenges, almost all students reported their ability to proficiently manipulate the system and utilize the functions to complete the assignment excellently and technically. For example, S8 answered: *“… The interfaces are quite simple for me to familiar with. I can get to know how to manipulate the system in a second. All of my classmates know how to handle it, I think. I just feel it is not smoothly to* log *in system. I think it is the internet problem … I will* log *out first and then* log *in again. If the system still cannot work, I will restart the system …”*. However, the frequent instability of the online homework system led to a black screen and server standby, compelling students to restart the system and redo the homework. Despite their attempts to seek assistance from customer service, students unanimously acknowledged that this issue not only consumed time but also demotivated them from continuing their homework tasks. What is worse, the sluggish speed of internet access added to their challenges, leading to prolonged waiting times and increased durations to complete online homework. For example, S9 commented: *“… I feel it is really annoying to handle this system when the internet speed is slow. What is worse, the system sometimes will crash down! It will kill me a lot of time when it's frozen …”*.

#### 5.2.4 Behavioral intention to complete online homework

Students conveyed their intention to use the online homework system in the interview as well. Among them, five students expressed their commitment to continuing the use of online homework. Four students emphasized that their inclination to continue using the system was triggered by enhanced academic performance. Additionally, the appeal of interesting content played a significant role in stimulating their interest in doing online homework. For example, S8 expressed: *“… compared to traditional homework, the content provided by the system is rich, vivid, and interesting … it is amazing to complete homework in such different ways … I am interested in doing online homework …”*.

Some students expressed a neutral opinion regarding online homework, indicating their inclination to engage might vary based on different circumstances. For example, S4 mentioned that he might consider doing online homework if he had time and in a good mood. On the other hand, S10 emphasized the usability of the system but he expressed concerns about their capability to engage in online homework without parental permission. He also indicated a preference for doing online homework, provided that his parents allowed him to use computers or mobile phones.

Several students indicated their unwillingness to continue using the system, citing various reasons. Their reasons included learning burden, unengaging content design, time wastage, unfriendly functions, as well as challenges associated with maintaining self-discipline while utilizing the online homework system. For example, S6 commented: *“… I would not choose online homework in the following days. Because I could not control myself to focus on doing homework while using the internet. Besides … the assessment function is wonderful, but I just do not want to be evaluated …”*.

## 6 Discussion

The purpose of online homework is to aid students in consolidating their learning and achieving mastery of knowledge. Attaining high academic performance in the exams serves as a manifestation of the benefits derived from online homework for student learning. In this study, the relationship between online homework completion and academic performance of Chinese secondary students based on post-test was analyzed. In the reports provided by the selected students, a positive correlation emerges, suggesting that the online homework system is associated with improvements in their academic performance. But the online homework completion variable explained only 26.1 % of the variance in post-test scores among students. In this case, the frequency of online homework completion does not dominantly account for better academic performance. It is possible that the students who get higher post-test scores may rely more on other factors, such as supervision from parents, self-regulation, higher pre-test scores, and so on [6,34,37,52,53].

The usability of the system and how users experience this system are often reflected by user experience [54]. Based on the qualitative data in this study, the act of doing online homework among students seems to be greatly influenced by the possibility of academic enhancement, because majority of students frequently reported their perception on academic performance enhancement. This result is in accordance with the Chinese educational culture [55]. In Asia, there is a strong emphasis on examinations and this approach is currently dominant in education. Exam results are heavily emphasized while pupils are learning [16]. The perception of academic enhancement among students with intention to use technology is justified to be most frequently mentioned herein. According to Creswell & Poth [56], the frequency of emerging themes in thematic analysis can be employed to assess the prevalence and influence of themes within qualitative data. Hence, the outstanding frequency of cognitive factors emerged in the qualitative data also may indicate that it is the most influential factor predicting students’ behaviors towards online homework.

Homework is commonly perceived as a monotonous and obligatory task that students are expected to complete [33]. As a format of homework, the inherent perception among students may have not evolved in the context of online homework. In this study, the mean of the frequency of online homework completion is only above 9, indicating that students do not have strong intention to complete this assignment. This is understanding because some students may perceive online homework as an extra burden for them, resulting in their frustration and disappointment in doing it [ 29, 57]. What is more, this study was conducted in a voluntary context, meaning that students obtain their own choices to do the assignments or not. Even though assigning homework provides students with practice opportunities that can promote their learning achievement, simply distributing homework does not guarantee its successful completion [58]. Rather, it is a student-side factor, such as student motivation [16] that determines them to do homework. Based on the interview data, many students who intended to complete homework via the system expressed their positive emotions towards the system. Their likeliness, enjoyment, interest, and preference towards online homework evoked them to do the homework [22,38,40,57,59,60]. This finding aligns with the conclusions by Cheng [36], Huang, Teo & Zhou [55], Huang [39], and Parker & Loudon [2], highlighting the impact of attitudes on students’ behavioral intention to use technology. What is more, the frequency of the emotional factors emerging in the interview data also suggests that this factor contributes a significant impact to the behaviors of doing homework for learning among students.

Students stressed the significance of interpersonal effect on their intention to use online homework. Specifically, when discussing the challenges that students had encountered during the learning procedure, they emphasized the crucial role of parental involvement in resolving these issues. These challenges predominantly encompassed disruptive learning environment and their procrastinating or gaming behaviours. The external distractions [24] as well as dishonest behaviors in homework completion among students [61] are widespread occurrences. Consequently, parents played a big role in encouraging their children to engage with online homework systems for educational purposes [62] and they were expected to create a conductive learning environment at home for students [16]. Notably, in the Chinese educational context, the inclination of children to use the system for learning was somewhat influenced by the support from parents and instructors [37].

The answers from the selected students also indicate that students potentially have adequate technology self-efficacy, enabling them to effectively use the online homework system properly. Furthermore, it is also possible that the interface design of the system must be easy to use for students. As some students reported, the interfaces were user-friendly. This is in line with the findings of a study conducted by Albelbisi & Yusop [63] and Williams [64], highlighting that the perceived ease of use in doing homework through systems may promote the students to adopt this homework format. Nonetheless, inadequate technical support within the system has resulted in an unsatisfactory user experience, leading to some students' reluctance to use the system. As Murphy, Roschelle, Feng & Mason [53] stressed, the availability of resources, as well as technical supports, significantly influences students’ intention to use online homework.

## 7 Conclusion

Homework serves as a familiar instructional method for students, providing them with the opportunity to reinforce and consolidate their learning. Similarly, online homework is a learning facilitator tool supported by technology to assist student learning. The primary goal of online homework is to assist students to experience better education. Hence, the outcome of using the systems via learning is instrumental in guiding students to pay more attention to online homework.

For RQ1, the relationship between online homework completion and academic performance among Chinese secondary students is positive, but the effect is small. It identifies the significance of online homework for student learning. Nevertheless, an excessive amount of repetitive homework may discourage students’ intention to do it either. Therefore, educators may assign homework based on practical and scientific educational principles to boost student learning. In addition, the low frequency of online homework completion in a voluntary context in this study also may indicate that enough supervision is essential for achieving better homework completion rate and academic performance. Otherwise, students may get addicted to procrastination or computer games, engendering a low instructional result.

The intention to accept, adopt, and use a learning system is instrumental to student usage of the system [54]. For RQ2, students have identified six key factors influencing their engagement in online homework, including cognitive factors, emotional factors, empathical factors, interpersonal factors, economical factors, and technological factors. It seems that human dimension, including cognitive factors and emotional factors, outweighs its influential role in the UX model based on the interview data of this study. Thus, to convince students that doing homework via the system is beneficial for their study is vital for instructors. What is more, students with interests and positive attitudes tend to have favorable experiences with online homework. Conversely, bored, tired and other negative moods may lead to a bad experience in doing online homework. Hence, educators should appropriately and skillfully control the difficulty level of the assignments, so as to evoke students’ enthusiasm for learning.

Additionally, social dimension also accounts for students' intention to completing homework via the system. So, establishing effective, friendly, and healthy communication between instructors, parents and students is also crucial for promoting system usage among students. Besides, students generally approve of the interface designs of the system, but they express dissatisfaction with the insufficient technical support when doing online homework. This highlights the need for shareholders to prioritize and ensure adequate facilitating condition to enable widespread implementation of the system. Furthermore, incorporating better feedback mechanisms within the system is recommended. For example, integrating a user-friendly assessment or evaluation function may alleviate students’ discontent with repetitive exercises.

To sum up, this study contributes to a better understanding of online homework completion and academic performance among Chinese secondary students for instructors. The exploration of the use of online homework on secondary students for English homework may expand the understanding of the effects of this facilitator on students' progress in the language learning domain as well. The cross-sectional research design of this study also brings the subsequent interview data to provide insights into tackling the low rate of homework completion among students. To stress, investigating the influential factors on students’ behaviors based on the UX model [32] is another novel attempt of this study, which may signify the importance of the user-end side when promoting technology for learning. Particularly, the emergence of the most influential role of cognitive and emotional factors in doing homework among students in a voluntary context is also important to offer reference to enhance students to learn independently.

## 8 Limitation and recommendations

The number of students and the duration are regarded as the restraining factors of this study. Furthermore, this study was conducted with cross-sectional design, hence, further studies may better explain the relationship between online homework completion and academic performance among secondary students through longitudinal data. Besides, the instructors should be considered and included in the study. Different roles of instructors may also contribute to a different influence on the academic achievement of the students. The interview reports might contain potential sources of bias in the collected data and subsequent findings. Additionally, the purposive sampling technique also restricts the generalization of the study results.

A fully quantitative research methodology is recommended to explore the same issue based on a validated measure. Analysis on factors influencing students’ behavioral intention to use the online homework system may be conducted as well. Some properties of user experience seem to be highlighted in this study, such as perceived of usefulness, hence, it is recommended that further studies may focus on the evaluation of different properties. What is more, studies on the comparison between different educational contexts are also significant for further investigation. Last but not least, future research endeavors may benefit from incorporating control variables such as background demographics and motivational beliefs to account for potential confounding factors and further elucidate the complex dynamics between online homework, academic performance, and other relevant variables.

## CRediT authorship contribution statement

**Liu Chen:** Writing – original draft, Conceptualization. **Su Luan Wong:** Investigation. **Shaoning Zeng:** Funding acquisition, Software. **Shwu Pyng How:** Writing – review & editing, Investigation.

## Informed consent statement

All participants were informed that consent to participant in the study and publish their data would be assumed on completion and submission of the study survey. Assent also was obtained from the legal guardians.

## Ethical statements

This research was reviewed and approved by the Ethics Committee for Research Involving Human Subjects of Universiti Putra Malaysia: JKEUPM-2022-739.

## Data availability

Sharing research data helps other researchers evaluate your findings, build on your work and to increase trust in your article. We encourage all our authors to make as much of their data publicly available as reasonably possible. Please note that your response to the following questions regarding the public data availability and the reasons for potentially not making data available will be available alongside your article upon publication.

## Declaration of competing interest

The authors declare that they have no known competing financial interests or personal relationships that could have appeared to influence the work reported in this paper.
