# Supplementary material for: Exploring Chinese secondary students’ behaviors towards online homework based on the UX model: Does homework completion relate to academic performance?
Source: Heliyon. 2024 Nov 22;10(23):e40472. doi: 10.1016/j.heliyon.2024.e40472 (PMC11636115; doi:10.1016/j.heliyon.2024.e40472)
Supplement: Multimedia component 1 [file mmc1.docx]

| Interview Protocol |
| --- |

Time of interview:

Date:

Place:

Interviewee:

Reminder: telling the interviewee about

1. The purpose of the study
2. The individuals and sources of data being collected
3. What will be done with the data to protect the confidentiality of the interviewee
4. How long the interview will take
5. Have the interviewee read and sign the consent form and
6. Test the video tools.

Questions for Interview:

1. How do you feel about online homework for your learning?
2. Tell me why you have such a feeling about online homework?
3. Give me an example from your experience to support how you feel about the online homework system, such as Ekwing?
4. What factors do you think impact you do online homework?
5. In what ways do you think the online homework system (ie., Ekwing) can be used for your learning?
6. Do you meet any difficulties in using the online homework system? If so, what can you do?
7. Will you continue to use the online homework system for your learning? Why?
8. If you can choose, will you do online homework or paper pencil homework? Why?
9. Would you like to add anything else?
10. Any questions for me?
